# Survival of Colon Cancer in a Population‐Based Cohort Study: A Comprehensive Analysis of Location of the Primary Tumor

**DOI:** 10.1002/cam4.72140

**Published:** 2026-08-19

**Authors:** Pauline Brindel, Evelyne Fournier, Pierre Olivier Chappuis, Thomas McKee, Mario Kreutzfeldt, Giacomo Puppa, Johan Ferrari, Jakob Nikolas Kather, Elisabetta Rapiti, Simone Benhamou

**Affiliations:** ^1^ Geneva Cancer Registry Institute of Global Health, University of Geneva Geneva Switzerland; ^2^ Oncogenetics Unit, Division of Precision Oncology Geneva University Hospitals Geneva Switzerland; ^3^ Division of Genetic Medicine Geneva University Hospitals Geneva Switzerland; ^4^ Department of Clinical Pathology Geneva University Hospitals Geneva Switzerland; ^5^ Department of Pathology and Immunology University of Geneva Geneva Switzerland; ^6^ Else Kroener Fresenius Center for Digital Health Faculty of Medicine and University Hospital Carl Gustav Carus, TUD Dresden University of Technology Dresden Germany; ^7^ Department of Medicine I Faculty of Medicine and University Hospital Carl Gustav Carus, TUD Dresden University of Technology Dresden Germany; ^8^ Medical Oncology, National Center for Tumor Diseases (NCT) University Hospital Heidelberg Heidelberg Germany; ^9^ Pathology & Data Analytics Leeds Institute of Medical Research at St James's, University of Leeds Leeds UK; ^10^ INSERM Unit 1018 Research Center on Epidemiology and Population Health Villejuif Île de France France

**Keywords:** biomarkers, colorectal cancer, molecular oncology, registry, survival

## Abstract

Disparities in survival between left‐ versus right‐sided colon cancer have been reported. We studied the role of sidedness on survival of colon cancer across tumor biomarkers subgroups in a population‐based cohort established using the Geneva Cancer Registry database. Our study included 3503 colon cancer cases diagnosed between 1985 and 2013. Right‐sided colon cancers (ICD‐O codes C18.0, C18.2–18.4) and left‐sided colon cancers (ICD‐O codes C18.5–18.7) were compared. Five‐year net survival was estimated using the Pohar‐Perme estimator. Flexible hazard models evaluated the association of survival with sidedness adjusted for patient, tumor, and treatment characteristics. Left‐sided colon cancers were more frequent than right‐sided colon cancers (53% vs. 47%). Right‐sided colon cancer cases were older and more often women, less frequently of stage I or well differentiated. Left‐sided colon cancer had a higher 5‐year net survival than right‐sided colon cancer (66% vs. 59%, *p* < 0.001). In a multivariable model, patients with a left‐sided tumor had a 25% reduction in 5‐year mortality compared with right‐sided tumors (excess hazard ratio [eHR]: 0.75, 95% confidence interval [CI]: 0.63–0.89, *p* = 0.001). This association remained significant only among stage IV while not among stages I‐III. Among a subgroup of 2438 cases, stratification on tumor molecular biomarkers confirmed the better prognosis of left‐sided colon cancer versus right‐sided colon cancer among microsatellite stable (MSS) tumors, tumors with BRAF wild‐type (BRAFwt), and iCMS2 tumors. This study shows the importance of sidedness for prognosis and treatment considerations in addition to tumor biomarkers. Further research is needed to assess the molecular mechanisms underlying these differences between left and right colon cancer to address the prognostic gap.

## Graphical abstract

Left‐sided colon cancer (LCC) displayed a better prognosis than right‐sided colon cancer (RCC) for microsatellite stable (MSS) tumors, tumors with BRAF wild‐type (BRAFwt), and iCMS2 tumors.

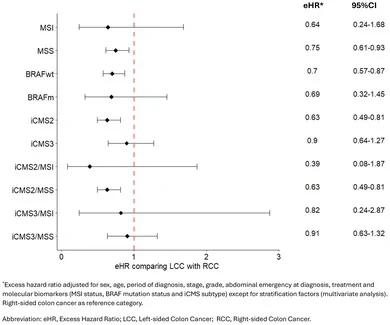

**Abbreviations**
ANOVA: analysis of variance
CI: confidence interval
CMS1: consensus molecular subtype 1
CMS2: consensus molecular subtype 2
CMS3: consensus molecular subtype 3
EGFR: epidermal growth factor receptor
eHR: excess hazard ratio
ESMO: European Society for Medical Oncology
HR: hazard ratio
ICD‐O: International Classification of Disease for Oncology
iCMS2: intrinsic‐consensus molecular subtype 2
iCMS3: intrinsic‐consensus molecular subtype 3
MSI: microsatellite instability
MSI‐H: microsatellite instability high
MSS: microsatellite stable
NOS: not otherwise specified
RCT: randomized clinical trial
Ref.: reference
Wt: wild‐type

## 1 Introduction

Right‐sided (proximal) colon cancer and left‐sided (distal) colon cancer display distinguishing phenotypes. In terms of molecular characteristics, right‐sided colon cancers display more often microsatellite instability (MSI), are hypermutated, present more often as BRAF mutant, and are more frequently CMS1, CMS3, and iCMS3, whereas left‐sided colon cancers are more frequently chromosomally instable, CMS2, and iCMS2 [1, 2]. The side of the primary tumor also plays a role in the prediction of treatment response, and the latest ESMO recommendations therefore integrate sidedness in addition to biomarkers to guide therapy with anti‐EGFR drugs for the treatment of metastatic colorectal cancer [3].

Survival of colon cancer has continuously improved over the past decades, with the introduction of screening programs, earlier detection of tumors, and the improvement of treatments, including the introduction of targeted therapies since 2000 [4]. Understanding the mechanisms involved in predicting the response to treatments of colorectal cancer in terms of survival outcomes has been a continuing process in the context of constant evolution and complexification of treatment options. Among potential prognostic factors, the role of sidedness on survival outcomes has gained more and more attention in oncological research. Several studies have consistently shown that survival of left‐colon cancer is significantly better that right‐sided colon cancer, across the various stages of the disease [5, 6, 7, 8, 9]. But population‐based studies on the role of sidedness in colon cancer survival are scarce, often with short‐term follow‐up and often lack information on adjusting factors such as molecular biomarkers or treatment [5, 6].

Our first objective was to evaluate the role of sidedness on survival in a population‐based study established using the Geneva Cancer Registry database. Our second objective was to explore the association among subgroups defined by the major tumor biomarkers.

## 2 Material and Methods

### 2.1 Data Source and Cohort Definition

The colorectal cancer cohort established using the Geneva Cancer Registry database was described previously [10]. This cohort is constituted of 5499 patients diagnosed between 1985 and 2013 with a primary invasive cancer of the colon, except appendix (International Classification of Diseases for Oncology, Third Edition [11], ICD‐O, C18.0, C18.2–C18.9), the rectosigmoid junction (ICD‐O, C19.9) and the rectum (ICD‐O, C20). Among patients who had multiple synchronous colorectal tumors (*n* = 81), identified as having the same date of diagnosis, only one tumor per individual was randomly selected and included in the study.

The present study focuses exclusively on colon cancer (*n* = 4244). Patients with colon cancer but no specified location were excluded (*n* = 549). Histology codes of included patients with colon cancer were as follows: 8000/3, 8010/3, 8070/3, 8140/3, 8144/3, 8200/3, 8201/3, 8210/3, 8211/3, 8220/3, 8221/3, 8255/3, 8260/3, 8261/3, 8262/3, 8263/3, 8380/3, 8410/3, 8440/3, 8480/3, 8481/3, 8490/3, 8510/3, 8560/3, 9990/3. To explore the long‐term outcomes, patients with a survival time after diagnosis of 30 days or less were excluded (*n* = 192). Accordingly, the final study population comprises 3503 patients (Figure S1). Ethical approval for this study was obtained from the Geneva Ethics Committee (Commission cantonale de la recherche scientifique de Genève (CCER), No. 2016‐00141). All patients were followed‐up for survival until December 31, 2021.

### 2.2 Variables of Interest

Variables of interest were age at diagnosis (< 60 years, 60–74 years, 75 years and over), sex (male, female), year of diagnosis (1985–1994, 1995–2004, 2005–2013), tumor stage in four groups (I, II, III, and IV) or two groups (I–III or IV), tumor differentiation (well, moderate, poor), and presentation with an abdominal emergency at diagnosis (Yes vs. No). The initial treatment received was considered in five subgroups: surgery alone, surgery with adjuvant chemotherapy, neoadjuvant chemotherapy with surgery, chemotherapy alone, and no treatment. Testing for tumor molecular markers was performed retrospectively according to study protocol. Detailed analysis methods are described in the Supporting Information. Tumor markers including microsatellite status, BRAF mutation, and iCMS subtype were categorized as follows: MSI versus MSS, BRAF mutant versus BRAF wild‐type, iCMS2 versus iCMS3. Lastly, the combination between microsatellite status and iCMS subtype was categorized as iCMS2‐MSS, iCMS2‐MSI, iCMS3‐MSS, and iCMS3‐MSI.

### 2.3 Statistical Analysis

We compared left‐sided colon cancer with right‐sided colon cancer, considering the splenic flexure as the frontier between right‐sided and left‐sided tumors: right‐sided colon cancer included locations from the cecum, ascending colon, hepatic flexure, and transverse colon (ICD‐O codes: C18.0, C18.2–18.4). Left‐sided tumors included tumors located at the splenic flexure, descending colon and sigmoid (ICD‐O codes: C18.5–18.7). A total of 1870 patients had a left‐sided colon cancer (53%) and 1633 a right‐sided colon cancer (47%).

Continuous data were expressed as mean and standard deviation or median and interquartile range. Chi‐square tests were performed to assess differences in categorical variables across primary tumor location and ANOVA was performed when considering continuous variables.

Patients were followed for survival from the date of diagnosis to the date of death or emigration or end of 2021, whichever came first. Net survival in a relative setting, defined as the survival that would be observed if cancer was the only possible cause of death, was calculated using the Pohar‐Perme estimator [12], and the necessary expected mortality rates by age, sex, and calendar year were extracted from the official Geneva life tables. Because expected numbers were too low among the 90‐year‐old patients and over, this group was excluded from the analyses. In a univariable analysis of net survival, comparisons according to categorical variables such as primary tumor location (left‐sided colon cancer vs. right‐sided colon cancer) used a log‐rank test. In a multivariable analysis, we used a flexible hazard model to estimate net survival according to the primary tumor location adjusted for sex, age, period of diagnosis, stage, grade, abdominal emergency at diagnosis, and treatment. To allow comparison with other studies, we also calculated 5‐year overall survival and 5‐year cause‐specific survival. We considered the overall survival time as the interval between the date of diagnosis and the date of death from any cause. In the cause‐specific setting, colon cancer specific survival time was defined as the interval between the date of diagnosis and the date of death from colon cancer, with the cause of death validated or revised from death certificates by the Geneva cancer registry.

Multiple imputations were performed to address variable missingness in the analysis before stratification [13]. Multiple imputations were run on datasets that included tumor sidedness, sex, age, period of diagnosis, stage, abdominal emergency at diagnosis, treatment, the Nelson–Aalen estimate of the cumulative hazard and the event indicator. Biomarkers were not included. Stage, treatment and abdominal emergency at diagnosis were the three variables with missing data. For stage and treatment, missing values were imputed using a multinomial regression model. For abdominal emergency at diagnosis, missing values were imputed using a logistic regression model. We imputed the missing data using the function ‘mice’ of the mice R‐package, conducting 20 imputations. A flexible hazard model, aligned with the main model used before imputation, including tumor sidedness, sex, age, period of diagnosis, stage, abdominal emergency at diagnosis and treatment, was applied to each of the imputed datasets before pooling of the results using the method developed by Antunes et al. [14].

Subgroup analyses were performed to explore the association with tumor sidedness separately according to stage (stages I–III and stage IV respectively) and status of microsatellite (MSI vs. MSS), BRAF mutation (BRAF mutant vs. BRAF wild‐type), and iCMS subtypes (iCMS2 vs. iCMS3). Interaction was tested between stage and primary tumor location and between each tumor marker and primary tumor location in a multivariable model. The statistical analyses were performed using Stata software, version 18 (StataCorp LLC), and R statistical software (https://www.cran.r‐project.org). Two‐tailed *p* < 0.05 was considered statistically significant.

## 3 Results

Patients and tumor characteristics according to left‐ or right‐sided colon cancer are presented in Table 1. No statistically significant difference in the repartition of colon cancer sidedness was observed over the study period (*p* = 0.6). Compared with left‐sided colon cancers, patients with right‐sided colon cancers were older (mean age at diagnosis: 72.8 vs. 68.8 years, *p* < 0.001) and more likely to be women (54.5% vs. 47.2%, *p* < 0.001) (Table 1).

**TABLE 1:** Patients and tumor characteristics according to tumor location among colon cancer patients.

	Right‐sided colon cancer	Left‐sided colon cancer	Total	*p*
	*N* = 1633	*N* = 1870		
Age (years)				
Mean (SD)	72.8 (12.4)	68.8 (12.1)	70.7 (12.4)	< 0.001
Follow–up time (years)				
Median (IQR)	4.1 (0.9 to 10.8)	6.4 (1.8 to 13.5)	5.1 (1.3 to 12.4)	< 0.001
Sex				
Male (%)	743 (45.5)	988 (52.8)	1731 (49.4)	< 0.001
Female (%)	890 (54.5)	882 (47.2)	1772 (50.6)	
Age				
≤ 59 years (%)	259 (15.9)	419 (22.4)	678 (19.4)	< 0.001
60–74 years (%)	535 (32.8)	781 (41.8)	1316 (37.6)	
≥ 75 years (%)	839 (51.4)	670 (35.8)	1509 (43.1)	
Period of diagnosis				
1985–1994 (%)	507 (31.0)	591 (31.6)	1098 (31.3)	0.6
1995–2004 (%)	547 (33.5)	648 (34.7)	1195 (34.1)	
2005–2013 (%)	579 (35.5)	631 (33.7)	1210 (34.5)	
Stage				
I (%)	193 (12.7)	372 (22.2)	565 (17.7)	< 0.001
II (%)	545 (36.0)	541 (32.2)	1086 (34.0)	
III (%)	408 (26.9)	379 (22.6)	787 (24.6)	
IV (%)	369 (24.4)	387 (23.0)	756 (23.7)	
Unknown	118	191	309	
Grade				
Well differentiated (%)	266 (18.6)	402 (24.8)	668 (21.9)	< 0.001
Moderately differentiated (%)	832 (58.3)	1063 (65.7)	1895 (62.2)	
Poorly differentiated (%)	330 (23.1)	153 (9.5)	483 (15.9)	
Unknown	205	252	457	
Microsatellite status				
MSI (%)	204 (18.4)	59 (5.1)	263 (11.6)	< 0.001
MSS (%)	905 (81.6)	1104 (94.9)	2009 (88.4)	
	524	707	1231	
BRAF status				
BRAF wild‐type (%)	981 (81.3)	1224 (96.8)	2205 (89.2)	< 0.001
BRAF mutated (%)	225 (18.7)	41 (3.2)	266 (10.8)	
	427	605	1032	
iCMS				
iCMS2 (%)	620 (52.1)	925 (74.2)	1545 (63.4)	< 0.001
iCMS3 (%)	570 (47.9)	321 (25.8)	891 (36.6)	
	443	624	1067	
iCMS/MSI status				
iCMS2‐MSS (%)	505 (46.8)	817 (71.9)	1322 (59.7)	< 0.001
iCMS2‐MSI (%)	51 (4.7)	28 (2.5)	79 (3.6)	
iCMS3‐MSS (%)	374 (34.7)	261 (23.0)	635 (28.7)	
iCMS3‐MSI (%)	149 (13.8)	31 (2.7)	180 (8.1)	
Abdominal emergency at diagnosis				
No (%)	973 (78.5)	1055 (74.6)	2028 (76.4)	0.020
Yes (%)	266 (21.5)	359 (25.4)	625 (23.6)	
Unknown	394	456	850	
Treatment				
Surgery alone (%)	1065 (68.4)	1235 (68.6)	2300 (68.5)	< 0.001
Surgery + adjuvant chemotherapy (%)	288 (18.5)	379 (21.1)	667 (19.9)	
Neoadjuvant chemotherapy + surgery (%)	17 (1.1)	48 (2.7)	65 (1.9)	
Chemotherapy alone (%)	32 (2.1)	12 (0.7)	44 (1.3)	
None (%)	155 (10.0)	126 (7.0)	281 (8.4)	
Unknown	76	70	146	

Right‐sided colon cancers presented less frequently with stage I (13% vs. 22%), were less frequently well differentiated tumors (18.6% vs. 24.8%, *p* < 0.001). In terms of biomarkers, right‐sided colon cancers were more frequently MSI (18.4% vs. 5.1%), exhibited more frequently a BRAF mutation (18.7% vs. 3.2%), and more often belonged to the iCMS3 subtype (47.9% vs. 25.8%) and to its iCMS3‐MSS subset (34.7% vs. 23.0%). Right‐sided colon cancers were less often diagnosed after an abdominal emergency (21.5% vs. 25.4%, *p* < 0.001) and were less often treated with a combination of surgery and chemotherapy than left‐sided colon cancers (19.7% vs. 23.7%, *p* < 0.001).

### 3.1 Results for Net Survival in a Relative Setting

**FIGURE 1:**
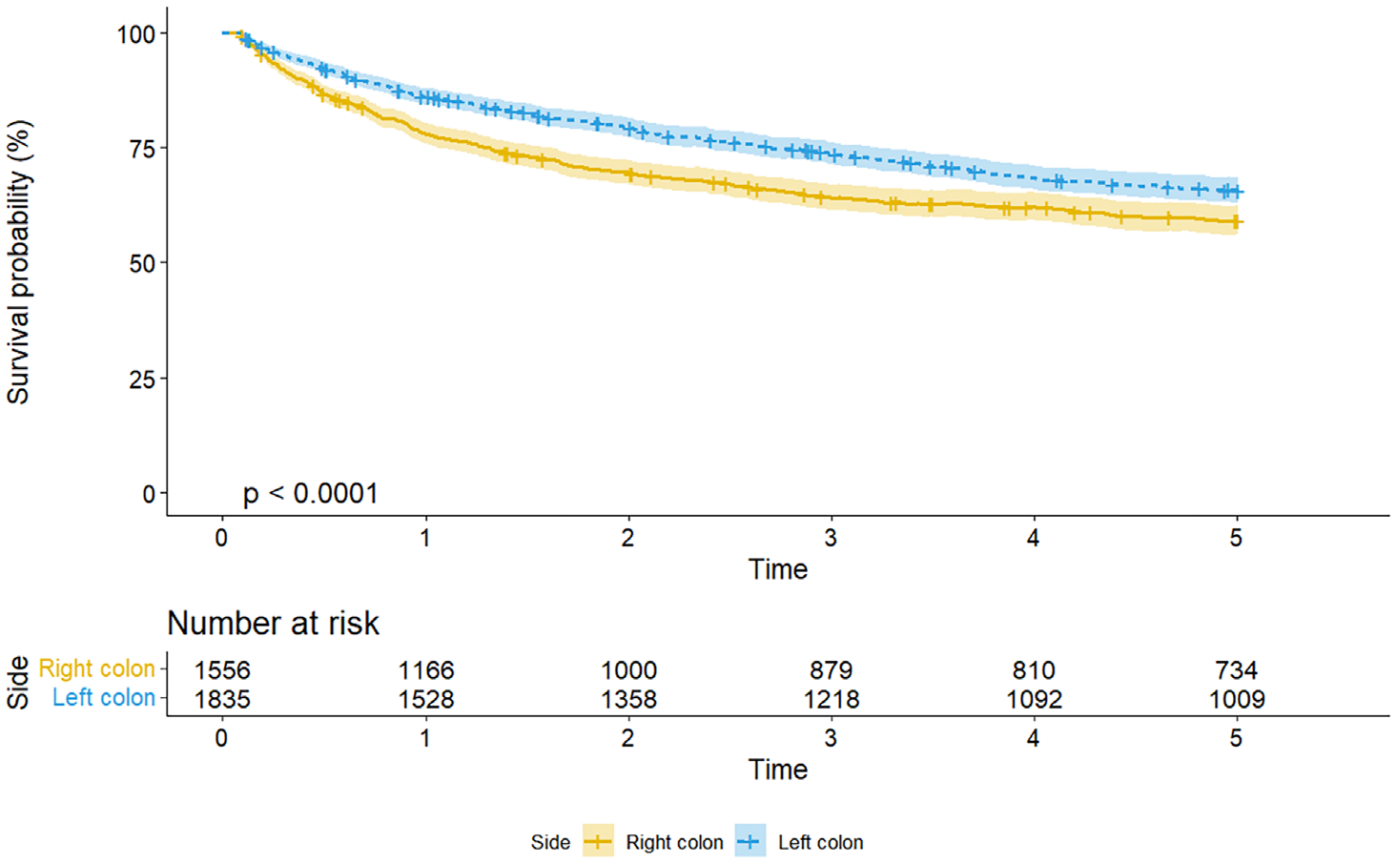
Net survival according to the side of the primary colon cancer, Geneva, 1985–2013.

**FIGURE 2:**
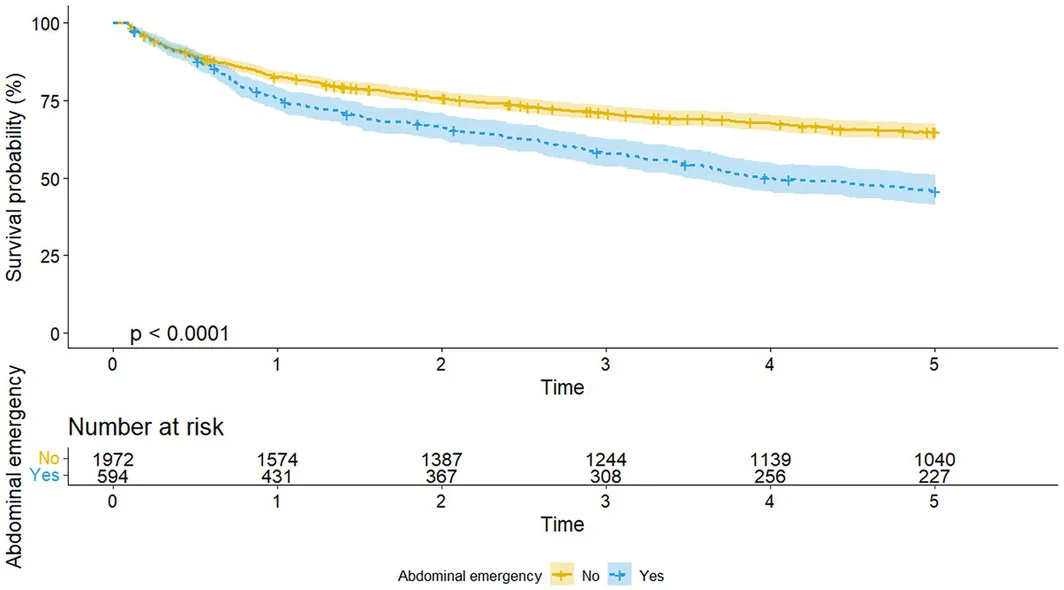
Net survival according to an abdominal emergency at diagnosis, Geneva, 1985–2013.

Five‐year net survival according to patient, tumor characteristics and treatment are reported in Table 2, Figures 1 and 2. Patients with left‐sided colon cancer had a 5‐year net survival of 66%, compared with 59% for those with right‐sided colon cancer (*p* log‐rank < 0.001) (Table 2 and Figure 1). Patients aged 75 years and older had a net survival of 57%, lower in comparison with 68% among patients under 60 years old. Net survival increased with more recent periods of diagnosis (69% in 2005–2013 compared with 56% in 1985–1994; *p* log‐rank < 0.001) (Table 2). Stage I or II tumors had a 5‐year net survival above 80%, respectively 96% and 87%, and decreased to 61% for stage III, and below 10% for stage IV (Table 2). Grade was also significantly associated with net survival, as 5‐year survival decreased with poorer degrees of differentiation (48% for poorly differentiated compared with 73% for well differentiated tumors; *p* log‐rank < 0.001). Patients presenting with an abdominal emergency at diagnosis had a worse survival with 46% compared with 65% for other patients (*p* log‐rank < 0.001) (Table 2 and Figure 2). Patients who underwent surgery without chemotherapy as initial treatment, which represented two thirds of the cohort, had a 5‐year net survival of 73%, while those who had surgery and chemotherapy had a 5‐year net survival of 56%. Most significant associations observed in the whole sample remained significant in both strata of stage (Table 2). The association with sidedness remained significant among patients with stage IV colon cancer but not among patients with stage I–III colon cancer.

**TABLE 2:** Five‐year net survival (after exclusion of 90‐year‐old and over patients).^a^

	All stages			Stage I–III			Stage IV		
	*N* (%)	5‐year net survival (95% CI)	*p*	*N* (%)	5‐year net survival (95% CI)	*p*	*N* (%)	5‐year net survival (95% CI)	*p*
Tumor location									
Left‐sided	1835 (54%)	66% (63%, 68%)	< 0.001	1273 (54%)	82% (79%, 85%)	0.12	380 (51%)	12% (8.9%, 16%)	< 0.001
Right‐sided	1556 (46%)	59% (56%, 62%)		1103 (46%)	79% (75%, 83%)		358 (49%)	7.5% (5.0%, 11%)	
Age									
≤ 59 years	678 (20%)	68% (64%, 71%)	< 0.001	466 (20%)	86% (82%, 89%)	0.009	169 (23%)	13% (9.0%, 20%)	< 0.001
60–74 years	1316 (39%)	67% (64%, 70%)		945 (40%)	83% (80%, 86%)		283 (38%)	11% (8.1%, 16%)	
≥ 75 years	1397 (41%)	57% (53%, 61%)		965 (41%)	75% (71%, 80%)		286 (39%)	6.0% (3.5%, 10%)	
Sex									
Male	1704 (50%)	63% (60%, 66%)	0.57	1190 (50%)	80% (77%, 84%)	0.87	380 (51%)	11% (7.7%, 15%)	0.37
Female	1687 (50%)	62% (59%, 65%)		1186 (50%)	81% (78%, 84%)		358 (49%)	8.9% (6.2%, 13%)	
Period of diagnosis									
1985–1994	1069 (32%)	56% (52%, 60%)	< 0.001	700 (29%)	73% (68%, 78%)	< 0.001	221 (30%)	4.9% (2.6%, 9.2%)	0.001
1995–2004	1156 (34%)	62% (59%, 66%)		816 (34%)	81% (77%, 85%)		270 (37%)	8.8% (5.8%, 13%)	
2005–2013	1166 (34%)	69% (66%, 73%)		860 (36%)	86% (82%, 90%)		247 (33%)	15% (11%, 21%)	
Stage									
I	558 (18%)	96% (92%, 100%)	—	558 (23%)	96% (92%, 100%)	—			
II	1055 (34%)	87% (83%, 90%)		1055 (44%)	87% (83%, 90%)				
III	763 (24%)	61% (56%, 65%)		763 (32%)	61% (56%, 65%)				
IV	738 (24%)	9.7% (7.7%, 12%)					738 (100%)	9.7% (7.7%, 12%)	
Grade									
Well differentiated	656 (22%)	73% (69%, 78%)	< 0.001	509 (23%)	82% (77%, 87%)	0.01	76 (13%)	11% (5.8%, 22%)	< 0.001
Moderately differentiated	1852 (62%)	67% (65%, 70%)		1414 (64%)	82% (79%, 85%)		376 (62%)	13% (10%, 18%)	
Poorly differentiated	468 (16%)	48% (43%, 54%)		296 (13%)	71% (65%, 79%)		156 (26%)	6.3% (3.2%, 12%)	
Abdominal emergency at diagnosis									
No	1972 (77%)	65% (62%, 68%)	< 0.001	1404 (77%)	84% (81%, 87%)	< 0.001	418 (73%)	8.9% (6.4%, 12%)	0.12
Yes	594 (23%)	46% (41%, 51%)		417 (23%)	60% (54%, 66%)		155 (27%)	11% (7.1%, 19%)	
Treatment									
Surgery only	2236 (69%)	73% (70%, 76%)	—	1824 (80%)	81% (78%, 84%)	0.007	258 (36%)	11% (7.3%, 16%)	—
Surgery + Chemotherapy	732 (23%)	56% (52%, 60%)		451 (20%)	80% (76%, 85%)		273 (39%)	15% (11%, 20%)	
Chemotherapy	41 (1.3%)	— (—, —)		1 (< 0.1%)			40 (5.6%)	— (—, —)	
None	240 (7.4%)	1.5% (0.5%, 4.6%)		13 (0.6%)			137 (19%)		
Microsatellite status									
MSI	256 (12%)	69% (62%, 78%)	0.1	215 (12%)	78% (70%, 87%)	0.9	35 (8%)	23% (12%, 45%)	0.9
MSS	1961 (88%)	63% (61%, 66%)		1510 (88%)	76% (73%, 80%)		406 (92%)	11% (9%, 15%)	
BRAF									
BRAF wild‐type	2162 (90%)	66% (63%, 69%)	0.41	1688 (89%)	79% (76%, 82%)	0.31	420 (90%)	13% (9.5%, 17%)	0.26
BRAF mutated	253 (10%)	65% (57%, 74%)		205 (11%)	75% (67%, 85%)		47 (10%)	16% (8%, 33%)	
iCMS									
iCMS2	1516 (64%)	64% (61%, 67%)	0.61	1167 (63%)	77% (74%, 81%)	0.91	320 (69%)	13% (9.7%, 18%)	0.005
iCMS3	863 (36%)	66% (62%, 71%)		690 (37%)	78% (74%, 83%)		147 (31%)	10% (6%, 17%)	
Combination iCMS and microsatellite status									
iCMS2‐MSS	1296 (60%)	61% (58%, 65%)	0.52	983 (59%)	75% (71%, 79%)	0.92	287 (66%)	13% (9%, 18%)	0.06
iCMS2‐MSI	77 (3.6%)	63% (51%, 77%)		63 (3.8%)	73% (60%, 89%)		12 (2.7%)	9.1% (2.1%, 40%)	
iCMS3‐MSS	613 (28%)	63% (59%, 69%)		479 (29%)	76% (71%, 82%)		115 (26%)	7% (3%, 14%)	
iCMS3‐MSI	171 (8.1%)	71% (62%, 82%)		148 (8.8%)	80% (70%, 91%)		23 (5.3%)	31% (15%, 63%)	

^a^ Net survival according to biomarkers was computed in the subgroup of patients among whom tumors were tested for such markers.

In a multivariable analysis in the total sample, tumor location was significantly associated with net survival, with left‐sided cancer patients having a 25% lower risk of mortality in comparison with right‐sided cancer patients (excess hazard ratio [eHR] 0.75; 95% confidence interval [CI], 0.63–0.89; *p* = 0.001) after adjustment for sex, age, period of diagnosis, stage, grade, abdominal emergency at diagnosis and treatment (Table S1). Net survival improved over time, with an almost 50% reduction in mortality between the first 10‐year period and the most recent time period of the cohort (eHR of 0.50 for 2005–2013 in comparison with 1985–1994). Stage was the strongest prognostic factor, with eHR of 17 for stage III reaching over 80 for stage IV compared with stage I. Patients presenting with an abdominal emergency at diagnosis had a significantly worse outcome than patients who did not (eHR 1.28; 95% CI, 1.07–1.53; *p* = 0.007). Patients who had undergone surgery and chemotherapy had better outcomes compared with those who had surgery only (eHR 0.63; 95% CI 0.51–0.77; *p* < 0.001). Absence of surgery was associated with an increased risk of mortality in comparison with surgical treatment (eHR 2.18; 95% CI, 1.32–3.61; *p* = 0.002 for chemotherapy only and eHR 4.62; 95% CI, 3.25–6.56; *p* < 0.001 for no treatment) (Table S1). We conducted multiple imputations to verify the robustness of our results obtained through complete case analysis. Sidedness remained significantly associated with survival after imputing stage, treatment and abdominal emergency.

In a multivariable analysis stratified on stage, the excess hazard ratio (eHR) comparing left‐sided tumors to right‐sided tumors was 0.73; 95% CI 0.59–0.91; *p* = 0.005 among stage IV versus 0.80; 95% CI 0.61–1.05; *p* = 0.11 for stages I–III (Table S1). Period of diagnosis was strongly associated with prognosis for both subgroups of cancer stage, with patients diagnosed in most recent periods showing better outcomes (eHR 0.44; 95% CI 0.30–0.63; *p* < 0.001 and 0.58; 95% CI 0.43–0.78; *p* < 0.001 for period 2005–2013 in comparison with 1985–1994, among stages I–III and among stage IV, respectively). Also, treatment was significantly associated with prognosis in both groups, with patients with surgery and chemotherapy having a better prognosis than patients with surgery only (eHR 0.61; 95% CI 0.42–0.88; *p* = 0.009 among stages I–III and eHR 0.59; 95% CI 0.45–0.77; *p* < 0.001 among stage IV). To explore further the role of metastasectomy among metastastic disease, we conducted analysis among the subgroup of stage IV disease with non‐missing information on metastasectomy (*n* = 561). In a multivariable model, patients with a metastasectomy had a significantly increased survival as compared with patients with no metastasectomy: eHR 0.48 95% CI (0.36–0.64) (*p* < 0.001). When adjusting for metastasectomy, the association of survival with sidedness was not significant in this subgroup (Table S6).

Sensitivity analyses were conducted in subgroups of patients for whom testing for tumor molecular markers had been performed. For the present study we focused on the microsatellite and BRAF status and the iCMS subtypes (Table 2, Figure 2 and Table S3).

Among the subsets of patients with molecular markers (all stages), we did not observe statistically significant differences of survival according to molecular markers, whether it be MSI status, BRAF mutation status or ICMS status (Table 2).

When stratifying on stage, iCMS3 tumors had a worse survival than iCMS2 tumors (10% vs. 13%, *p* = 0.005) among stage IV (Table 2).

We then studied the association of sidedness with survival in strata of molecular markers, adjusted for other factors in separate multivariable models, and tested interactions between tumor location and type of biomarker. Results are presented in Table S3. Association with sidedness remained statistically significant among MSS tumors after adjusting for BRAF‐mutation status and iCMS subtypes (eHR, 0.75 for left‐sided vs. right‐sided; 95% CI, 0.61–0.95, *p* = 0.007). The association with sidedness also remained statistically significant among patients whose tumors exhibited the BRAF wild‐type (eHR, 0.70 for left‐sided vs. right‐sided; 95% CI, 0.57–0.87, *p* = 0.001). No significant interaction was observed between MSI status and sidedness (*p* = 0.60) nor between BRAF‐mutation status and sidedness (*p* = 0.55) (Table S3). Among patients with iCMS2 subtype tumor, left‐sided tumors had a statistically significant increased survival (eHR, 0.63; 95% CI, 0.49–0.81) compared with right‐sided tumors, whereas no association was observed among iCMS3 (Table S3). There was a significant interaction between sidedness and iCMS subtypes before adjustment on BRAF‐mutation status and microsatellite status, and nearly statistically significant when adjusting on those tumor markers. Finally, in the subgroup of patients with iCMS2‐MSS tumors, left‐sided tumors had a 37% reduction in mortality in comparison with right‐sided colon cancers.

### 3.2 Results for Overall Survival and Disease‐Specific Survival in the Whole Cohort

The 5‐year overall survival for patients with left‐sided colon cancer was 56% compared with 47% for patients with right‐sided colon cancer (*p* < 0.001) (Table S2).

The 5‐year disease‐specific survival for patients with left‐sided colon cancer was 67% compared with 59% for patients with right‐sided colon cancer (*p* < 0.001) (Table S2).

## 4 Discussion

In this population‐based cohort study of 3503 patients diagnosed with colon cancer, left‐sided cancer patients had a 25% lower risk of mortality in comparison with right‐sided cancer patients after adjusting for patient, tumor and treatment characteristics. The association remained statistically significant among stage IV but not among stages I–III patients. Furthermore, in the subgroups of MSS tumors, BRAF wild‐type (BRAFwt) tumors and iCMS2 tumors, there was a clear association of the left‐side location with a better prognosis. Our results also suggest an interaction between iCMS subtype status and sidedness, that is, the fact that left‐side location is associated with better survival in iCMS2 tumors whereas no such association is observed for iCMS3 tumors.

A prior study in Geneva including 3396 patients with colon cancer diagnosed between 1980 and 2006 showed that cause‐specific survival was higher among left‐sided colon cancers than among right‐sided colon cancers (HR, 0.81; 95% CI, 0.72–0.90, *p* < 0.001) [7]. Tumor sidedness was associated with survival outcomes in several studies and two meta‐analyses. In a meta‐analysis of Petrelli et al., overall survival was significantly associated with sidedness (HR, 0.73; 95% CI 0.69–0.78 for left‐sided vs. right‐sided tumors among stage IV tumors and HR, 0.84, 95% CI 0.79–0.89 among stages I–III tumors, *p* < 0.001 for subgroups difference) [5]. In the meta‐analysis of Ha et al., patients with left‐sided colon cancer also had a better net survival (in a cause‐specific setting) than right‐sided colon cancer patients, but only among patients with stage III colon cancers [6]. On the contrary, for less advanced stages represented by stages I and II, survival was significantly better for right‐sided cancers. Other studies, not included in the meta‐analyses, suggested an interaction with stage. In a population‐based study conducted in Norway, in which survival estimates were provided across four time periods spanning from 1977 to 2016, the distant disease group was the only subgroup for which left‐sided colon cancer showed consistently better outcomes than right‐sided colon cancer across time periods. Among localized or regional disease, in contrast, the association with sidedness was most often not statistically significant [8]. In another population‐based study conducted in Belgium, left‐sided colon cancer had a better 5‐year relative survival with stronger association among stages III and IV [9]. Finally, in a population‐based study focused on metastatic colorectal cancers, left‐sided colon cancer had a significantly better survival than right‐sided colon cancer among patients with synchronous liver metastasis with a 3‐year net survival of 16.0% (95% CI, 14.0%–18.2%) for left‐sided colon and 9.4% (95% CI, 7.8%–11.2%) for right colon cancer (*p* < 0.001) [15].

Some authors have suggested that this advantage of survival among left‐sided versus right‐sided cancers could be related to lead time bias and earlier screening, while better treatment could also be implicated. In Geneva, organized colorectal cancer screening programs started recently in 2019, after the inclusion of the patients in the cohort. Therefore, we could not study the effect of screening on the role of sidedness on survival in our study.

One of the possible explanations for the association of survival with sidedness could be related to varying molecular features such as the routinely analyzed biomarkers, namely microsatellite instability (MSI) and BRAF mutation status. Tumors have been shown to display different biomarkers along the gut in a way that the frequency of detection of BRAF mutation or MSI in colon cancer decreases from the proximal to the distal extremity of the colon. As a consequence, MSI is more frequent among right‐sided cancers than left‐sided cancers and this molecular trait is a marker of good prognosis across all stages [16]. BRAF mutation on the other hand is associated with a worse prognosis, but that could be overridden by MSI‐linked prognosis [17].

To consider the possible confounding effect of MSI and BRAF on the association of survival with sidedness, a consortium of studies including more than 13,000 cases of colorectal cancer was conducted with the objective to study the role of tumor location on cause‐specific survival in strata of MSI‐H or non MSI‐H status [16]. This study highlighted a significant interaction between the primary location of the tumor and MSI status. In the non MSI‐H subgroup, cause‐specific survival increased from the cecum to the sigmoid with a significant trend, while an opposite trend was observed among MSI‐H (*p* interaction = 0.001) [16]. Our results also partly support these findings since the MSS left‐sided colon cancer subgroup in our study also had a better prognosis than right‐sided colon cancer (eHR, 0.73; 95% CI, 0.60–0.89), *p* = 0.002 (Table S3). However, unlike the consortium study, the interaction between MSI status and sidedness was not statistically significant in our study. Our sample size was limited for the study of the association of sidedness and survival within MSI‐H tumors. Similarly, we observed an association of sidedness and survival among BRAF‐wt tumors, which was also observed in the consortium study [16].

MSI‐H and BRAF do not explain all differences in prognosis between right and left‐sided cancers. Several proposed signatures such as the consensus molecular subtypes [18] and the more recently identified epithelial signature that provide useful refinements for the classification of colorectal cancer and with proven prognostic value and prediction of treatment, have been shown to be related to sidedness [2]. The recently identified epithelial signature distinguishes five subtypes based on the intrinsic subtype (iCMS2 or iCMS3), microsatellite instability status (MSS or MSI‐H) and fibrosis. According to this classification, iCMS2 is more often observed among left‐sided tumors and associated with good prognosis while iCMS3 is more often observed among right‐sided tumors and is associated with a worse prognosis [2]. In our cohort, left‐sided colon cancer displayed a predominance of iCMS2 tumors compared with iCMS3 tumors (74% vs. 26%). When we conducted analysis by strata of intrinsic subtype (iCMS2 vs. iCMS3), the association of left‐sided cancer with a better prognosis was maintained in the iCMS2 subgroup, unlike among iCMS3. There was a statistically significant interaction between sidedness and iCMS subtype before adjustment on other tumor molecular biomarkers and nearly significant after adjusting for those markers. Tumor location could be a surrogate of other factors that could explain differences observed among iCMS2 tumors. We hypothesize that since iCMS3 already have the worst prognosis, iCMS3 overrides the role of sidedness, while there may be more variety of different prognoses in iCMS2 tumors among which tumor location influences prognosis.

### 4.1 Strengths and Limitations

#### 4.1.1 Strengths

Our study has several strengths. One of the main strengths was the possibility to conduct the analysis in a large sample of patients for whom identification of biomarkers could be performed in order to study the association of survival with sidedness in most prevalent subgroups of tumor biomarkers with sufficient statistical power. Furthermore, thanks to a thorough review of each individual case by the interviewers, using each case's linked documents available at the Geneva Cancer Registry, valuable prognostic variables such as treatment are nearly exhaustive and the quality of the data is high. Consequently, the association of survival with sidedness is adjusted on several factors (such as age, period of diagnosis, and tumor and treatment characteristics) to minimize confounding bias. This was possible through the performance of a multivariable analysis using multivariable models adapted for net survival to estimate excess hazard ratios accounting for adjusting factors.

Second, as a population‐based study including all patients registered in the Geneva cancer registry that covers all institutions in the area, the population is unselected on the contrary to hospital‐based studies or studies conducted through a reanalysis of randomized controlled trial populations. Therefore, selection biases are limited, reinforcing the external validity of our results.

Third, when other studies mainly reported overall survival and cause‐specific survival, we provided the Pohar‐Perme estimator as we focused on net survival in a relative setting to better approach the mortality arising only from colon cancer, disregarding the influence of other causes of death [12]. This method allows comparison with studies conducted in other populations where mortality from other causes could vary substantially.

#### 4.1.2 Limitations

This study has several limitations. First, although we had information on treatment and the type of therapy for the most recent periods, very few patients received biomarker guided therapy, such as targeted therapies. This is due to missing data and also relates to the period of inclusion of the cases (1985–2013), during which colorectal cancer treatment did not involve such biomarker‐guided integration of biologicals. Indeed, these treatments were introduced after 2005 and initially only for metastatic colorectal cancers. As a consequence, the initial treatments received by patients in our cohort may not reflect current practices, which may limit the generalizability of our findings. For example, MSI is currently considered for its predictive value for use of immune checkpoint inhibitors [3]. Introduced in 2017, this therapy is considered as a game changer for the management of MSI‐high advanced CRC [3].

Second, we were not able to detail treatment options in order to consider the role of metastasectomy among the whole sample of metastatic diseases, because of missing data. Although the association of survival with sidedness was not significant when adjusting for metastasectomy in a sensitivity analysis, this result needs to be interpreted with caution because of the high number of missing information for metastasectomy and also the additional performed tests.

Third, KRAS mutation status was not characterized for the tumors included in our study, although this is a marker of poor prognosis [19]. However, several studies that included this marker did not identify any interaction between this tumor biomarker and tumor location when studying survival [9, 16].

A fourth limitation resides in the multiple biomarkers defined subgroups in which we conducted our analysis of the role of sidedness on survival, since this implied multiple testing with a risk for false‐positive results. As a consequence, some of our subgroup results need to be interpreted with caution and should be considered more as exploratory.

Finally, our study was retrospective and observational and as a result, some information remained incomplete even after thorough revision of existing documents attached to the already registered data. However, it is unlikely that this information bias was in relation to sidedness. A non‐negligible proportion of colon cancer cases (13%) in our cohort were classified as location “Colon NOS” for Not Otherwise Specified, which led to their exclusion from the cohort. Among included cases, missing variables were limited to under 10% for stage, but around 24% for abdominal emergency management at diagnosis. We conducted multiple imputations to verify the robustness of our results obtained through complete case analysis.

## 5 Conclusion

Although progress in the management of colon cancer has been made over decades, right‐sided colon cancer has worse outcomes than left‐sided colon cancer in terms of survival, particularly among patients with metastatic disease. This study indicates the importance of sidedness for prognosis and treatment considerations in addition to tumor biomarkers. These results confirm previous findings that right‐sided colon cancer should be considered as a particular entity that needs to be addressed [7]. Further research is needed to continue to explore the epidemiology and the molecular mechanisms underlying this difference in prognosis to drive the adaptation of cancer prevention, early detection and management to address this disease.

## Author Contributions

**Thomas McKee:** conceptualization, methodology, validation, investigation, resources, data curation, writing – original draft, writing – review and editing, visualization, supervision, project administration, funding acquisition. **Pierre Olivier Chappuis:** conceptualization, methodology, validation, investigation, writing – original draft, writing – review and editing, visualization, supervision, project administration, funding acquisition. **Giacomo Puppa:** conceptualization, methodology, validation, investigation, resources, data curation, writing – review and editing, supervision, project administration. **Johan Ferrari:** conceptualization, methodology, validation, investigation, resources, data curation, writing – review and editing, supervision, project administration. **Pauline Brindel:** conceptualization, methodology, software, validation, formal analysis, investigation, data curation, writing – original draft, writing – review and editing, visualization, supervision, project administration. **Evelyne Fournier:** conceptualization, methodology, software, validation, formal analysis, investigation, data curation, writing – original draft, writing – review and editing, visualization, supervision, project administration. **Simone Benhamou:** conceptualization, methodology, validation, investigation, resources, writing – original draft, writing – review and editing, visualization, supervision, project administration, funding acquisition. **Elisabetta Rapiti:** conceptualization, methodology, validation, investigation, resources, writing – original draft, writing – review and editing, visualization, supervision, project administration, funding acquisition. **Jakob Nikolas Kather:** conceptualization, methodology, validation, investigation, resources, data curation, writing – review and editing, supervision, project administration. **Mario Kreutzfeldt:** conceptualization, methodology, validation, investigation, resources, data curation, writing – original draft, writing – review and editing, supervision, project administration, visualization.

## Funding

The study was supported by grants from the Swiss National Science Foundation (320030‐163342/1), the Swiss Cancer League (KFS‐3932‐08‐2016‐R and KFS‐5243‐02‐2021), and the Ligue Genevoise contre le Cancer (subvention 1813).

## Ethics Statement

Ethical approval for this study was obtained from Geneva Ethics Committee (Commission cantonale de la recherche scientifique de Genève (CCER), No. 2016‐00141).

## Conflicts of Interest

J.N.K. declares consulting services for Bioptimus, France; Panakeia, UK; AstraZeneca, UK; and MultiplexDx, Slovakia. Furthermore, he holds shares in StratifAI, Germany, Synagen, Germany, and Ignition Lab, Germany; has received an institutional research grant by GSK; and has received honoraria by AstraZeneca, Bayer, Daiichi Sankyo, Eisai, Janssen, Merck, MSD, BMS, Roche, Pfizer, and Fresenius.

## Supporting information


**Figure S1:** Selection of eligible cases for the analyses.
**Table S1:** Factors associated with excess mortality in patients with colon cancer (multivariate analysis)^†^.
**Table S2:** Five‐year overall and disease‐specific survival.
**Table S3:** Association of primary tumor location with net survival stratified by stage and tumor biomarkers (multivariate net survival analysis).
**Table S4:** Distribution of metastasectomy and location of metastasis in case of metastasectomy, among stage IV colon cancers (*n* = 756).
**Table S5:** Net survival among stage IV colon cancer, according to metastasectomy status.
**Table S6:** Excess hazard ratios, multivariable analysis among stage IV colon cancers.

## Data Availability

The data that support the findings of this study are available from the corresponding author upon reasonable request.
