# Supplementary material for: Survival of Colon Cancer in a Population‐Based Cohort Study: A Comprehensive Analysis of Location of the Primary Tumor
Source: Cancer Med. 2026 Aug 19;15(8):e72140. doi: 10.1002/cam4.72140 (PMC13489814; doi:10.1002/cam4.72140)
Supplement: Supplementary file 1 [file CAM4-15-e72140-s001.docx]

Supplementary Figure 1: Selection of eligible cases for the analyses.

**Excluded (n=1804)**

**- C20 Rectum (n=1255)**

**- C18.9 Colon, NOS (n=39)**

**- C19.9 Rectosigmoid junction (n=503)**

**- Overlapping lesion of colon (C18.8) (n=7)**

**Geneva population-based**

**colorectal cancer cohort (n=5499)**

**Patients with right or left-sided colon cancer (n=3695)**

**Excluded (n=192)**

**- survival time<30 days**

**Right or left-sided colon cancer, survival ≥30 days and included in the analysis of overall and cancer specific survival (n=3503)**

**-Left sided location, n=1870 (53%)**

**-Right-sided location, n=1633 (47%)**

**Excluded (n=202)**

**- Age ≥90 years old**

**Right or left-sided colon cancer, survival ≥30 days, <90 years old and included in net survival analysis before adjustment on biomarkers (n=3391)**

**Excluded (n=953)**

**- Unresected tumors (n=302)**

**- All biomarkers missing (n=651)**

**Patients with right or left-sided colon cancer and survival ≥30 days and <90 years old and included in net survival analysis adjusted for biomarkers and stratified on biomarkers (n=2438)**

Supplementary Table 1: Factors associated with excess mortality in patients with colon cancer (multivariate analysis) †.

|  |  | Total | All |  | Stages I-III |  | Stage IV |  |
| --- | --- | --- | --- | --- | --- | --- | --- | --- |
| Factors |  | N | Excess Hazard Ratio eHR (95% CI) | p-value | Excess Hazard Ratio eHR (95% CI) | p-value | Excess Hazard Ratio eHR (95% CI) | p-value |
| Tumor location | Right-sided | 1,542 | Reference |  | Reference |  | Reference |  |
|  | Left-sided | 1,830 | 0.75 (0.63–0.89) | 0.001 | 0.80 (0.61–1.05) | 0.11 | 0.73 (0.59–0.91) | 0.005 |
| Sex | Male | 1,673 | Reference |  | Reference |  | Reference |  |
|  | Female | 1,699 | 0.88 (0.75–1.04) | 0.13 | 0.80 (0.61–1.05) | 0.11 | 0.93 (0.75–1.14) | 0.48 |
| Age | ≤59 years | 678 | Ref |  |  |  |  |  |
|  | 60–74 years | 1,313 | 1.01 (0.81–1.27) | 0.92 | 1.39 (0.93–2.08) | 0.10 | 0.90 (0.68–1.18) | 0.44 |
|  | ≥75 years | 1,381 | 1.50 (1.17–1.93) | 0.002 | 2.15 (1.40–3.29) | <0.001 | 1.24 (0.91–1.71) | 0.18 |
| Period of diagnosis | 1985–1994 | 1,061 | Reference |  | Reference |  | Reference |  |
|  | 1995–2004 | 1,149 | 0.81 (0.66–0.99) | 0.037 | 0.68 (0.49–0.93) | 0.02 | 0.93 (0.71–1.21) | 0.57 |
|  | 2005–2013 | 1,162 | 0.50 (0.40– 0.63) | <0.001 | 0.44 (0.30–0.63) | <0.001 | 0.58 (0.43–0.78) | <0.001 |
| Stage | I | 554 | Reference |  | Reference |  | - |  |
|  | II | 1,046 | 3.76 (1.25–11.36) | 0.02 | 3.48 (1.06–11.4) | 0.04 | - |  |
|  | III | 757 | 17.29 (5.87–50.91) | <0.001 | 14.9 (4.6–48.1) | <0.001 | - |  |
|  | IV | 738 | 85.44 (29.12–250.67) | <0.001 |  |  |  |  |
| Grade | Well differentiated | 652 | Reference |  | Reference |  | Reference |  |
|  | Moderately differentiated | 1,841 | 0.82 (0.64–1.05) | 0.13 | 0.95 (0.65–1.38) | 0.78 | 0.76 (0.55–1.06) | 0.10 |
|  | Poorly differentiated | 466 | 1.45 (1.09 –1.92) | 0.01 | 1.60 (1.03–2.48) | 0.04 | 1.37 (0.95–1.99) | 0.09 |
| Abdominal emergency at diagnosis | No | 591 | Reference |  | Reference |  | Reference |  |
|  | Yes | 1,960 | 1.28 (1.07–1.53) | 0.007 | 2.09 (1.60–2.73) | <0.001 | 0.93 (0.74–1.19) | 0.57 |
| Treatment | Surgery | 2,218 | Reference |  | Reference |  | Reference |  |
|  | Surgery + Chemotherapy | 729 | 0.63 (0.51–0.77) | <0.001 | 0.61 (0.42–0.88) | 0.009 | 0.59 (0.45–0.77) | <0.001 |
|  | Chemotherapy | 42 | 2.18 (1.32–3.61) | 0.002 |  |  | 1.71 (1.00–2.93) | 0.05 |
|  | None | 240 | 4.62 (3.25–6.56) | <0.001 |  |  | 4.19 (2.89–6.06) | <0.001 |

† Excess hazard regression model with baseline hazard described by the exponential of a cubic B-spline with knots at 1 and 5 years, variables included were tumor location, sex, age, period of diagnosis, stage (among all and among stage I-III), grade, abdominal emergency at diagnosis and treatment.

Supplementary Table 2: Five-year overall and disease-specific survival.

|  | N | Overall survival |  | Disease specific survival |  |
| --- | --- | --- | --- | --- | --- |
|  |  | 5 y (95% CI) | p | 5 y (95% CI) | p |
| **Tumor location** |  |  |  |  |  |
| Right-sided | 1,633 | 47% (45%, 50%) | <0.001 | 59% (57%, 62%) | <0.001 |
| Left-sided | 1,870 | 56% (54%, 59%) |  | 67% (65%, 70%) |  |
| **Age** |  |  |  |  |  |
| ≤59 years | 678 | 66% (63%, 70%) | <0.001 | 71% (68%, 75%) | <0.001 |
| 60–74 years | 1,316 | 61% (58%, 64%) |  | 69% (67%, 72%) |  |
| ≥75 years | 1,509 | 38% (36%, 41%) |  | 55% (52%, 58%) |  |
| **Sex** |  |  |  |  |  |
| Male | 1,731 | 52% (50%, 54%) | 0.9 | 65% (62%, 67%) | 0.08 |
| Female | 1,772 | 52% (50%, 55%) |  | 62% (60%, 65%) |  |
| **Period of diagnosis** |  |  |  |  |  |
| 1985–1994 | 1,098 | 45% (42%, 48%) | <0.001 | 55% (52%, 58%) | <0.001 |
| 1995–2004 | 1,195 | 53% (50%, 56%) |  | 64% (61%, 67%) |  |
| 2005–2013 | 1,210 | 58% (55%, 61%) |  | 71% (68%, 74%) |  |
| **Stage** |  |  |  |  |  |
| 1 | 565 | 83% (80%, 86%) | <0.001 | 97% (96%, 99%) | <0.001 |
| 2 | 1,086 | 71% (69%, 74%) |  | 85% (82%, 87%) |  |
| 3 | 787 | 51% (48%, 55%) |  | 62% (59%, 66%) |  |
| 4 | 756 | 8.4% (6.6%, 11%) |  | 11% (8.5%, 13%) |  |
| **Grade** |  |  |  |  |  |
| Well differentiated | 668 | 61% (58%, 65%) | <0.001 | 76% (72%, 79%) | <0.001 |
| Moderately differentiated | 1,895 | 57% (55%, 59%) |  | 67% (65%, 70%) |  |
| Poorly differentiated | 483 | 39% (35%, 44%) |  | 47% (43%, 52%) |  |
| **Abdominal emergency** |  |  |  |  |  |
| No | 2,028 | 54% (52%, 56%) | <0.001 | 65% (63%, 68%) | <0.001 |
| Yes | 625 | 38% (35%, 42%) |  | 49% (45%, 54%) |  |
| **Treatment** |  |  |  |  |  |
| Surgery only | 2,300 | 60% (58%, 62%) | <0.001 | 74% (72%, 75%) | <0.001 |
| Surgery and Chemotherapy | 732 | 52% (48%, 56%) |  | 56% (53%, 60%) |  |
| Chemotherapy | 44 |  |  |  |  |
| None | 281 | 1.1% (0.3%, 3.3%) |  | 4.9% (2.4%, 10.0%) |  |

Supplementary Table 3: Association of primary tumor location with net survival stratified by stage and tumor biomarkers (multivariate net survival analysis).

| Factors |  | N | Excess Hazard Ratio  eHR (95% CI) ^†^ | p-value | p*-*inter | Excess Hazard Ratio  eHR (95% CI) ^‡^ | p-value | p*-*inter |
| --- | --- | --- | --- | --- | --- | --- | --- | --- |
| **All** |  |  |  |  |  |  |  |  |
| Tumor location | Right-sided | 1,542 | Reference |  |  | Reference |  |  |
|  | Left-sided | 1,830 | 0.75 (0.63–0.89) | 0.001 |  | 0.75 (0.61–0.91) | 0.005 |  |
| **Stage** |  |  |  |  |  |  |  |  |
| **Stages I-III** |  |  |  |  | 0.66 |  |  | 0.35 |
| Tumor location | Right-sided | 1,089 | Reference |  |  | Reference |  |  |
|  | Left-sided | 1,268 | 0.80 (0.61–1.1) | 0.11 |  | 0.85 (0.63–1.14) | 0.28 |  |
| **Stage IV** |  |  |  |  |  |  |  |  |
| Tumor location | Right-sided | 358 | Reference |  |  | Reference |  |  |
|  | Left-sided | 380 | 0.69 (0.55–0.85) | <0.001 |  | 0.65 (0.49–0.86) | 0.003 |  |
| **MSI status** |  |  |  |  |  |  |  |  |
| MSS |  |  |  |  | 0.50 |  |  | 0.60 |
| Tumor location | Right-sided | 862 | Reference |  |  | Reference |  |  |
|  | Left-sided | 1,085 | 0.73 (0.60–0.89) | 0.002 |  | 0.75 (0.61–0.93) | 0.007 |  |
| MSI |  |  |  |  |  |  |  |  |
| Tumor location | Right-sided | 195 | Reference |  |  | Reference |  |  |
|  | Left-sided | 59 | 0.70 (0.28–1.75) | 0.45 |  | 0.64 (0.24–1.68) | 0.36 |  |
| **BRAF mutation status** |  |  |  |  |  |  |  |  |
| BRAFm |  |  |  |  | 0.50 |  |  | 0.55 |
| Tumor location | Right-sided | 209 | Reference |  |  | Reference |  |  |
|  | Left-sided | 41 | 0.70 (0.35–1.43) | 0.33 |  | 0.69 (0.32–1.45) | 0.32 |  |
| BRAFwt |  |  |  |  |  |  |  |  |
| Tumor location | Right-sided | 943 | Reference |  |  | Reference |  |  |
|  | Left-sided | 1,204 | 0.72 (0.59–0.88) | 0.002 |  | 0.70 (0.57–0.87) | 0.001 |  |
| **iCMS** |  |  |  |  | 0.035 |  |  | 0.06 |
| iCMS2 |  |  |  |  |  |  |  |  |
| Tumor location | Right-sided | 597 | Reference |  |  | Reference |  |  |
|  | Left-sided | 912 | 0.60 (0.47–0.76) | <0.001 |  | 0.63 (0.49–0.81) | <0.001 |  |
| iCMS3 |  |  |  |  |  |  |  |  |
| Tumor location | Right-sided | 538 | Reference |  |  | Reference |  |  |
|  | Left-sided | 314 | 0.95 (0.69–1.31) | 0.76 |  | 0.90 (0.64–1.3) | 0.56 |  |
| **Combination of iCMS subtype and microsatellite status** |  |  |  |  | NA |  |  | NA |
| iCMS2-MSS |  |  |  |  |  |  |  |  |
| Tumor location | Right-sided | 486 | Reference |  |  | Reference |  |  |
|  | Left-sided | 805 | 0.62 (0.48–0.79) | <0.001 |  | 0.63 (0.49–0.81) | <0.001 |  |
| iCMS2-MSI |  |  |  |  |  |  |  |  |
| Tumor location | Right-sided | 49 | Reference |  |  | Reference |  |  |
|  | Left-sided | 28 | 0.37 (0.08–1.7) | 0.20 |  | 0.39 (0.08–1.9) | 0.24 |  |
| iCMS3-MSS |  |  |  |  |  |  |  |  |
| Tumor location | Right-sided | 350 | Reference |  |  | Reference |  |  |
|  | Left-sided | 254 | 0.90 (0.63–1.3) | 0.58 |  | 0.91 (0.63–1.3) | 0.63 |  |
| iCMS3-MSI |  |  |  |  |  |  |  |  |
| Tumor location | Right-sided | 142 | Reference |  |  | Reference |  |  |
|  | Left-sided | 31 | 0.54 (0.12–2.4) | 0.42 |  | 0.82 (0.24–2.9) | 0.76 |  |

^†^ Excess hazard ratio adjusted for sex, age, period of diagnosis, stage, grade, abdominal emergency at diagnosis, treatment except for stratification factors (multivariate analysis).

^‡^ Excess hazard ratio adjusted for sex, age, period of diagnosis, stage, grade, abdominal emergency at diagnosis, treatment with further adjustment for MSI status, BRAF mutation status and iCMS subtypes, except for stratification factors (multivariate analysis).

Supplementary table 4. Distribution of metastasectomy and location of metastasis in case of metastasectomy, among stage IV colon cancers (n=756).

| Characteristics | Right-sided colon cancer  N=369 | Left-sided colon cancer  N=387 | p value |
| --- | --- | --- | --- |
| Metastasectomy |  |  |  |
| No | 207 (78%) | 202 (69%) | 0.02 |
| Yes | 60 (22%) | 92 (31%) |  |
| Unknown | 102 | 93 |  |
| Location of metastasis |  |  | 0.035 |
| Liver | 35 (58%) | 73 (80%) |  |
| Lymphatic node | 2 (3.3%) | 3 (3.3%) |  |
| Brain | 2 (3.3%) | 0 (%) |  |
| Other location | 21 (35%) | 15 (16.5%) |  |
| Unknown | 309 | 296 |  |

Supplementary table 5. Net survival among stage IV colon cancer, according to metastasectomy status.

|  | N | 5-year net survival (95% CI) | p value |
| --- | --- | --- | --- |
| Metastasectomy |  |  |  |
| No | 409 | 2.9% (1.6%-5.3%) | <0.001 |
| Yes | 152 | 24% (18%-33%) |  |

Supplementary table 6. Excess hazard ratios, multivariable analysis among stage IV colon cancers

| Factors |  | Excess Hazard Ratio eHR (95% CI) | p-value |
| --- | --- | --- | --- |
| Tumor location | Right-sided | Reference |  |
|  | Left-sided | 0.93 (0.72-1.19) | 0.5 |
| Sex | Male | Reference |  |
|  | Female | 0.99 (0.78-1.25) | 0.9 |
| Age | ≤59 years | Reference |  |
|  | 60–74 years | 0.92 (0.67-1.25) | 0.6 |
|  | ≥75 years | 1.27 (0.88-1.83) | 0.2 |
| Period of diagnosis | 1985–1994 | Reference |  |
|  | 1995–2004 | 0.82 (0.61-1.10) | 0.2 |
|  | 2005–2013 | 0.61 (0.44-0.84) | 0.003 |
| Grade | Well differentiated | Reference |  |
|  | Moderately differentiated | 0.59 (0.41-0.84) | 0.005 |
|  | Poorly differentiated | 1.24 (0.82-1.88) | 0.3 |
| Abdominal emergency at diagnosis | No | Reference |  |
|  | Yes | 0.88 (0.67-1.16) | 0.4 |
| Surgery | No | Reference |  |
|  | Yes | 0.29 (0.21-0.41) | <0.001 |
| Chemotherapy | No | Reference |  |
|  | Yes | 0.63 (0.78-0.83) | <0.001 |
| Metastasectomy | No | Reference |  |
|  | Yes | 0.48 (0.36-0.64) | <0.001 |

† Excess hazard regression model with baseline hazard described by the exponential of a cubic B-spline with knots at 1 and 5 years, variables included were tumor location, sex, age, period of diagnosis, grade, abdominal emergency at diagnosis, surgery, chemotherapy and metastasectomy.

**Biomarker staining and image analysis**

Immunohistochemical single stainings were performed on a Benchmark Ultra system (Roche Diagnostics, USA) according to manufacturer’s recommendations. Immunohistochemical multiplex stainings were performed on 4-μm thick sections from FFPE blocks using the fully automated Discovery Ultra system (Roche Diagnostics, USA), which carried out baking, deparaffinization, cell conditioning, staining, IHC, counterstaining, and titration. Antigen retrieval was performed using CC1 for 72 minutes, followed by incubation with a 2% normal goat serum solution at 37°C for 32 minutes and a peroxidase blocking step. First layer: Mouse monoclonal anti-CDX2 antibody (CDX2-88, MU392S-UC, BioGenex) was incubated for 60 minutes at 37°C at a 1:100 dilution. A system anti-Mouse HQ, followed by anti-HQ-HRP and a Purple detection kit, was used. A peroxidase neutralization step was then performed. Second layer: Rabbit monoclonal anti-MUC5AC antibody (MUC5AC (E3O91), 61193, Cell Signaling) was incubated for 60 minutes at 37°C at a 1:800 dilution. OmniMap Rabbit HRP was applied for 24 minutes, followed by a Green detection kit. A denaturation step using CC2 at 100°C for 24 minutes was then performed. Third layer: Mouse monoclonal anti-MUC2 antibody (SC-515032, LABFORCE) was incubated for 60 minutes at 37°C at a 1:400 dilution. UltraMap Mouse AP was applied for 24 minutes, followed by a Yellow detection kit for 44 minutes. Final layer: Mouse monoclonal anti-CD8 antibody (C8/144B, M7103, DAKO) was incubated for 60 minutes at 37°C at a 1:50 dilution. An amplification system (Amplification Kit) was applied, followed by OmniMap Mouse HRP and DAB detection. Hematoxylin (8 minutes) and Bluing Reagent (4 minutes) were used for counterstaining.

Stained slides were digitized at a resolution of 0.25 um/px on a Panoramic P1000 whole slide scanner (3DHistech, Hungary). Image analysis was performed using custom scripts developed in Visiopharm Oncotopix software (Visiopharm, Denmark). Quantification of BRAF expression followed the common guidelines. MSI status was evaluated by the combination of the expression patterns of PMS2:MLH1 and MSH2:MSH6, respectively. The classification of iCMS2 and iCMS3 was performed using the abundance of epithelial Muc2, Muc5 and Cdx2 marker expression.

BRAF quantification

BRAF quantification: Original image (left) stained with anti-BRAFV600E DAB (brown) and analyzed image (right). Colors correspond to detected tissue (blue outlines), detected tumor epithelium (red) and intraepithelial DAB-positive pixels (green). Scale bar =100um

iCMS classification:

ICMS classification: Tumor epithelium (green) and stroma (yellow) was detected. CD8 T cells were quantified for both compartments (inset, upper panel). Muc5 and Cdx2 positive areas were quantified within the tumor epithelium only (inset, lower panel). Scale bar = 100um, scale bar inset = 40um

**Antibodies:**

MLH1 (M1) ref: 760-5091 Roche
MSH2 (G219-1129) ref: 760-5093 Roche
MSH6 (SP93) ref: 760-5092 Roche
PMS2 (A16-4) ref: 760-5094 Roche

BRAF (VE1) ref: 760-5095 Roche
CD8 (C8/144B) ref: M7103 Dako

Muc2 ref: SC-515032, Labforce

Muc5AC (E3O91) ref: 61193, Cell Signaling

CDX2-88 ref: MU392S-UC, BioGenex
